# How could nanobiotechnology improve treatment outcomes of anti-TNF-α therapy in inflammatory bowel disease? Current knowledge, future directions

**DOI:** 10.1186/s12951-021-01090-1

**Published:** 2021-10-29

**Authors:** Piotr Eder, Aleksandra Zielińska, Jacek Karczewski, Agnieszka Dobrowolska, Ryszard Słomski, Eliana B. Souto

**Affiliations:** 1 Department of Gastroenterology, Dietetics and Internal Diseases, Poznan University of Medical Sciences, Przybyszewskiego Street 49, 60-355 Poznan, Poland; 2 Institute of Human Genetics, Polish Academy of Sciences, Strzeszyńska 32, 60-479 Poznan, Poland; 3 Department of Environmental Medicine, Poznan University of Medical Sciences, 61-701 Poznan, Poland; 4 CEB–Centre of Biological Engineering, University of Minho, Campus de Gualtar, 4710-057 Braga, Portugal

**Keywords:** Anti-TNF-α antibodies therapy, Inflammatory bowel diseases, Lipid nanoparticles, Oral drug delivery

## Abstract

Despite significant advances in therapeutic possibilities for the treatment of inflammatory bowel disease (IBD) in recent years, there is still a big room for improvement. In particular, biological treatment can induce not only clinical remission but also mucosal healing of the gastrointestinal tract. Among these therapeutic molecules, anti-tumor necrosis factor-alpha (anti-TNF-α) antibodies were the first to revolutionize treatment algorithms in IBD. However, due to the parenteral route of administration and systemic mode of action, TNF-α blockers are characterised by high rates of immunogenicity-related loss of response and serious adverse events. Moreover, intravenous or subcutaneous therapy is not considered patient-friendly and requires occasional, direct contact with healthcare centres. To overcome these limitations, several attempts have been made to design oral pharmaceutical formulations of these molecules. It is hypothesized that oral anti-TNF-α antibodies therapy can directly provide a targeted and potent anti-inflammatory effect in the inflamed gastrointestinal tissues without significant systemic exposure, improving long-term treatment outcomes and safety. In this review, we discuss the current knowledge and future perspectives regarding different approaches made towards entering a new era of oral anti-TNF-α therapy, namely, the tailoring of biocompatible nanoparticles with anti-TNF-α antibodies for site-specific targeting to IBD. In particular, we discuss the latest concepts applying the achievements of nanotechnology-based drug design in this area.

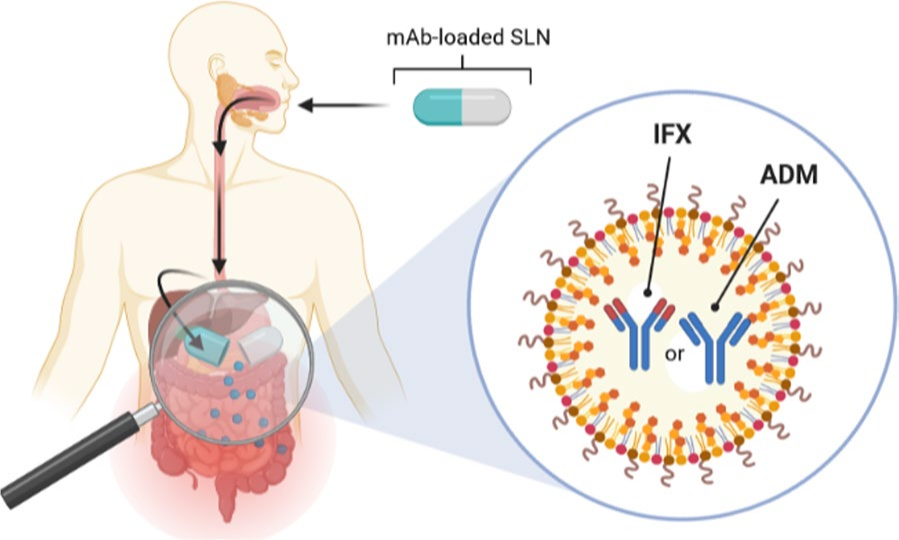

## Background

Inflammatory bowel disease (IBD) represents chronic, disabling, and incurable gastrointestinal disorders of unknown origin. There are two main disease entities classified as IBD—Crohn’s disease (CD) and ulcerative colitis (UC). Approximately seven million people suffer from IBD globally [1]. Furthermore, the incidence of these diseases is still increasing worldwide. The clinical course of IBD can be very diverse, leading in a significant proportion of those primarily young patients to irreversible bowel damage. Thus, IBD is not only a growing medical problem but also a social one [2]. Therefore, actions aimed at improving the results of IBD treatment should be considered a priority.

The main goal of IBD therapy is to heal the inflamed gastrointestinal tract, achieving steroid-free remission, and protect the patients from irreversible bowel damage and disability [3]. The therapeutic armamentarium includes various pharmacological agents, including aminosalicylates (5-ASA), immunosuppressants (thiopurines, methotrexate), steroids, small molecule drugs (tofacitinib, ozanimod), and biological drugs [4]. The former therapeutic category, in particular, has revolutionized treatment strategies in IBD in the last 20 years. Anti-tumor necrosis factor-α (anti-TNF-α) antibodies and newer groups of monoclonal antibodies (mAbs) directed against α4β7 integrin (vedolizumab) or interleukin-12 and -23 (IL-12/23) (ustekinumab) are characterized by the highest anti-inflammatory potential [4].

Despite the apparent advances in the quality of care in IBD, there is still a broad range of unanswered questions regarding how to improve the therapeutic outcomes further. One of the new strategies undertaken in this area is developing new pharmaceutical formulas of already used and effective drugs to overcome their known limitations, including immunogenicity and adverse events. In this paper, we present the current knowledge and future directions in terms of the oral administration of mAbs, as these biological molecules have been described as the most suitable approach to decorate nanoparticles for site-specific targeting. In particular, we discuss the application of the newest achievements in nanotechnology-based drug design in this area.

## Limitations of currently available pharmaceutical formulations of anti-TNF-α antibodies

**Fig. 1:**
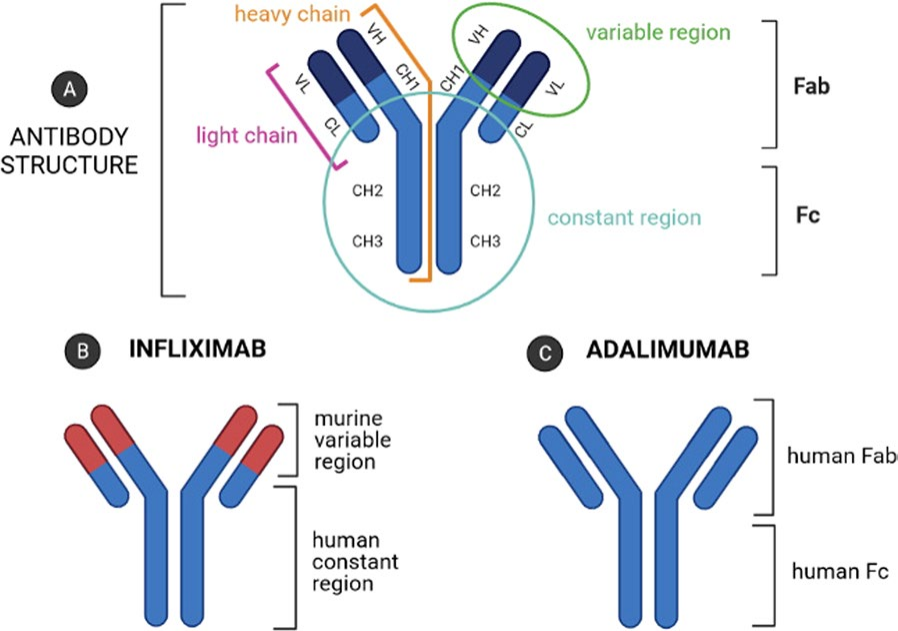
Schematic representation of IgG antibody structure (**A**) consisting of a shorter light chain (pink) and a long heavy chain (orange), the constant region (Fc, marked in blue), and its antigen-binding fragment (Fab, marked in green). VL: light chain variable region; VH: heavy chain variable region; CL: a constant region of the light chain; (CH1, CH2, CH3): regions of the heavy chain labeled 1, 2, 3, respectively. Schematic structure of infliximab (**B**) and adalimumab (**C**) [own drawing]

Infliximab (IFX) and adalimumab (ADM) are the two most widely used TNF-α inhibitors [5, 6]. IFX is a chimeric human-mouse IgG1 mAb, while ADM is a fully human IgG1 mAb (Fig. 1).

Both drugs are administered parenterally—IFX intravenously or subcutaneously, ADA—subcutaneously [5–7]. These routes of administration result in a systemic mode of action. On the one hand, this can be beneficial for those IBD patients with the most severe disease course. On the other hand, it can result in specific and possibly life-threatening side effects, encompassing systemic infections, allergic reactions, decompensation of cardiac failure, and many others [5, 6]. Moreover, the parenteral route of administration is associated with immunogenicity. According to frequent reports, approximately 70% of patients receiving IFX and 40% of those receiving ADM develop neutralizing anti-drug antibodies (ADAs) [8]. This phenomenon is believed to be responsible for high rates of primary (~ 30%) and secondary (~ 50%) non-response to anti-TNF-α inhibitors in IBD [8–10].

Several steps have been undertaken to improve these results. One of the therapeutic strategies adopted depends on a combination of a TNF-α inhibitor together with an immunomodulator (a combo therapy) [11]. Multiple studies, including one of the largest and robust ones—the SONIC trial, provided evidence for higher remission rates, improvement in mucosal healing, and lower incidence of a secondary non-response among patients receiving dual treatment, probably by reducing the frequency of ADAs generation [8, 11, 12]. Another strategy is a personalized therapeutic approach by performing reactive or proactive drug monitoring [8]. It is hypothesized that the measurement of IFX or ADM serum levels and ADAs can help optimize the dosing of anti-TNF-α agents, which could improve the long-term effectiveness of the therapy. Unfortunately, despite all these efforts, a significant proportion of IBD patients treated with TNF-α inhibitors still experience therapeutic failure [8–10].

Another possible limitation of the current forms of anti-TNF-α therapy, associated with the parenteral route of drug administration, is a need for regular and direct contact with healthcare providers, including the need for hospitalizations. This limitation is relevant mainly for patients receiving intravenous IFX injections. While subcutaneous administration of ADM or IFX is relatively easy, and most patients can do it without any assistance, there is still a need to undergo training supervised by a professional healthcare provider and visit the healthcare centre occasionally. This limitation is fundamental in the context of restrictions and recommendations for maintaining social distance related to the coronavirus disease-19 (COVID-19) pandemic [7, 13, 14]. Moreover, both parenteral routes of administration can be associated with pain, stress, and discomfort, therefore they are not considered fully patient-friendly [15].

Considering all the limitations of currently available pharmaceutical formulations of anti-TNF-α antibodies discussed above, it is of high importance to search for new solutions to improve therapeutic outcomes and their safety. Developing a unique formula of orally administered TNF-α inhibitors seems to fulfil all criteria for being a significant step forward in the quality of care in IBD. Table 1 summarizes the potential advantages and limitations of parenteral (intravenous and subcutaneous) and oral routes of administration of anti-TNF-α-acting molecules in IBD.

**Table 1:** Advantages and limitations of intravenous/subcutaneous (currently in use) and oral (under investigation) administration of anti-tumor necrosis factor-α-acting molecules in inflammatory bowel disease

Intravenous or subcutaneous route		Oral route	
Advantages	Limitations	Advantages	Limitations
The modifiable onset of action (IV—immediate, SC—immediate or modified-release)	Invasive and uncomfortable for the patient	Simplicity of administration	The relatively low onset of action
By definition, avoidance of the first-pass effect and 100% bioavailability	Drug administration usually needs a healthcare professional support and guidance	No need for technical healthcare professional support	Bioavailability below 100% and to some extent unpredictable pharmacokinetics due to possible interaction with gastrointestinal fluid content
A high systemic drug concentration is achievable	Usually need for visiting hospital or outpatient (with different frequency)	No need for regular visiting hospital and/or outpatient clinic	First-pass effect (does not refer to the idea of local oral administration aimed at targeting the inflamed intestinal wall)
Systemic mode of action (if needed)	The drug needs to be prepared in sterile conditions (IV)	Comfortable and painless application	Difficult to use in the case of uncooperative and unconscious patients
Possible in uncooperative and unconscious patients	In case of false dosing, higher risk of overdosing	Improved safety issues	Contraindicated in patients with intestinal obstruction
Possible in vomiting patients and the case of intestinal obstruction	Systemic mode of action (if not needed)	Targeted mode of action directly in the inflamed intestinal wall	Possible interference with food products
No direct interference with food	Specific adverse effects (IV—thrombophlebitis, catheter-related bloodstream infection; tissue necrosis—SC)	No or limited systemic exposure (if not needed)	Limited systemic mode of action (if needed)
	Immunogenicity and risk of secondary loss of response	Low immunogenicity and lower risk of secondary loss of response	Possibility of overdosing
	Costs	The modifiable onset of release and action (depending on drug design)	
		Biocompatibility and biodegradability (“eco-friendliness”)	
		Improvement in drug stability (especially in the case of lipid nanoparticles)	
		Costs	

Interestingly, there is some evidence on the potential oral application of TNF-α-neutralizing antisense oligonucleotides or small interfering RNAs [16]. However, all of these experiments were conducted using animal models of colitis. In contrast to that, the largest body of evidence is currently available for oral anti-TNF-α mAbs. Moreover, one should bear in mind that only anti-TNF-α mAbs (given parenterally) are used in the therapy of human IBD. That is why it seems that the concept of oral administration of this type of biological agents is the most promising one with the biggest potential to be implemented in clinical practice.

## Current knowledge on orally administered monoclonal antibodies in IBD

Currently, mAbs are administered mainly parenterally. Oral delivery of proteins remains a significant challenge. However, due to dynamic advances in drug design and chemistry, the possibilities to develop this specific pharmaceutical formula of different therapeutic molecules have improved significantly. Several approaches have been proposed to increase the stability of orally administered antibodies against the acidic and protease-rich environment of the gastrointestinal tract [17]; these include formulating them in liposomes, coating them with polymers, and genetic engineering of gastro-resistant forms [18, 19]. The high molecular weight of these drugs may also compromise their absorption into the periphery, which results in a relatively low risk of significant systemic exposure to the therapeutic molecule [17].

Ochi et al. were the first to show that oral administration of a mAb—anti-CD3 effectively suppressed experimental autoimmune encephalomyelitis [20]. In 2010, Ilan et al. described that oral dosing of anti-CD3 (OKT3) mAbs in healthy volunteers is safe, does not develop neutralizing antibodies, and is biologically effective [21]. These studies substantiate the feasibility of oral administration of mAbs-composed formulations in different clinical settings.

As a consequence of these developments, interest in the oral administration of mAbs for targeted intestinal drug delivery in IBD has also increased in recent years [22, 23]. For example, the aforementioned oral form of anti-CD3 (OKT3) mAb has also been tested in the treatment of moderate-to-severe UC [24]. Anti-CD3 molecule—muromonab-CD3 specifically binds to the T3 antigen complex (CD3) on human T lymphocytes and modulates several T-cell-mediated immune response functions. It has been registered to treat allograft rejections after transplantation. Originally, it is administered parenterally, however, due to a high risk of severe adverse effects, its application in other immune-mediated conditions is limited. To overcome these limitations, an oral form of anti-CD3 was developed. This molecule in a murine T-cell-induced colitis model significantly altered cytokine responses and showed high efficacy in reducing the inflammatory activity [25]. Subsequently, a small open-label pilot study was performed assessing the utility of oral OKT3 therapy in UC of moderate-to-severe activity [24]. The therapy was well-tolerated, and no serious adverse events were noted. The drug promoted an anti-inflammatory response when assessed on the gene expression levels in peripheral mononuclear cells. Due to a relatively small number of participants, it was impossible to determine the therapeutic clinical benefit. Unfortunately, the development of OKT3 is no longer actively promoted in the United States, and further analyses are not available [24]. Nevertheless, it was shown to be entirely feasible to develop an oral form of biologically active mAb without the risk of its inactivation in the gastrointestinal tract. Oral administration induced targeted, local immunomodulatory effect with low systemic drug exposure and was characterized by a good safety profile.

The studies on the efficacy and tolerability of OKT3 in IBD represented a fascinating approach. However, it investigated a novel therapeutic molecule administered in a completely new, experimental way. It seems more feasible and clinically relevant to design a novel orally administered formulation but containing a drug with a known and precisely proven efficacy, like TNF-α inhibitors.

The first attempts to develop an oral form of TNF-α blockers were undertaken by Worledge et al. already in 2000 [26]. The authors demonstrated that oral administration of avian anti-TNF-α antibodies significantly decreased inflammation in colonic tissues in a rat model of chemically-induced colitis. Interestingly, these effects were more pronounced when compared to sulfasalazine and dexamethasone. Another interesting concept in this area was proposed by Vandenbroucke et al. [27]. They developed a strain of *Lactococcus lactis* secreting monovalent and bivalent TNF-neutralizing nanobodies. The authors demonstrated that oral administration of these bacteria resulted in a local, colonic secretion of anti-TNF molecules, which induced anti-inflammatory effects in a dextran sulfate sodium (DSS) chronic colitis in mice. Bhol et al. [75] proposed AVX-470—a polyclonal antibody directed against TNF-α—as a candidate for IBD oral administration. The authors confirmed its anti-inflammatory properties both in in vitro and in vivo experiments in different animal models of IBD. The authors designed a randomized controlled trial in patients with moderate to severe UC in the next step [28, 29]. The study showed a dose-dependent beneficial trend in terms of clinical, endoscopic response and inflammatory biomarkers in patients receiving the drug compared to placebo. Orally administered AVX-470 was safe and well-tolerated with minimal systemic circulation absorption and no immunogenicity induction. Maurer et al. [30] formulated a 5 mg IFX tablet by incorporating the mAb into a sugar glass matrix based on oligosaccharide inulin and coated by a colon-specific ColoPulse release system. They showed that this formulation was stable in a long-term observation period. After 16 months, a mean 83% biological activity of the drug closed in vials and stored at 25 °C was detected. The authors further validated their results, showing the high stability and potency of ColoPulse-IFX compared to fresh IFX stock [31]. Since the production process of this formulation was validated, clinical trials are the final step of the ColoPulse-IFX investigation.

Another concept was proposed by Crowe et al. [32]. In their experimental model, a V565 domain of anti-TNF-α antibody was used, which is believed to be biologically active and resistant to intestinal proteases. They demonstrated that V565 was highly influential in neutralizing both the soluble and membrane-bound form of TNF-α. What is more, the investigated molecule was biologically stable after incubation with proteolytic enzymes and when exposed to intestinal and fecal supernatants. The V565 domain achieved a high concentration in the colonic tissue and stool after oral administration in a murine model of DSS-induced colitis. The authors also detected its levels in the serum, providing evidence for some penetration of the molecule through the inflammatory-disrupted intestinal wall. The same group developed enteric-coated mini-tablets of V565 resistant to gastric content and dissolving in the small intestine [33]. These experimental molecules were then orally administered in cynomolgus monkeys. The authors confirmed the small intestine as the site of mini-tablets dissolution and detected V565 in the stool, providing evidence of drug survival in the gastrointestinal tract after oral dosing. At the same time, it was shown that systemic exposure to V565 was very low. The same group showed in a small human IBD study that oral V565 mini-tablets were protected in the stomach and then gradually released in the intestines achieving a high local concentration and providing a decrease in inflammatory markers in colonic biopsies taken from UC patients after 7 days of treatment [34].

In 2021 results from a phase 2a clinical trial have been published showing that administration of OPRX-106—a novel oral TNF-α-blocking molecule is effective and safe in patients with mild-to-moderate UC [35]. OPRX-106 is a lyophilized *Nicotiana tabacum* (BY2) tobacco plant expressing recombinant TNFR2-Fc fusion protein. In this study, twenty-five UC patients were enrolled in an open-label manner to receive two different doses of OPRX-106 for 8 weeks. At the end of the evaluation, 67% and 28% of patients experienced clinical response and remission, respectively. This was accompanied by the reduction in fecal inflammatory markers and improvement in colonic histological scores. In parallel to clinical assessment, the authors conducted further research on the potential mechanisms of action of the investigated molecule. While they detected no significant absorption of OPRX-106 into the systemic circulation, they were able to show an increase in a CD4+ CD25+ FoxP3 subset of anti-inflammatory, suppressor T lymphocytes. One possible explanation for this phenomenon is the interaction of anti-TNF-α-acting molecule via Fc-receptor with a subgroup of CD14+/HLA-DR+ cells [36]. On the one hand, this interaction results in the production of IL-10—an anti-inflammatory cytokine. On the other hand, it promotes suppressor cells like Tregs or regulatory macrophages and NK cells.

Another important observation was the reduction of IL-6 and interferon-gamma levels after administration of OPRX-106 [35]. IL-6 seems to be the crucial cytokine in IBD responsible for the resistance of CD4+ T helper cells in inflammatory infiltrates to proapoptotic stimuli [36]. This phenomenon is mediated by the transmembrane TNF (tmTNF) interaction on monocytic cells with TNFR2 on CD4+ helper cells. The administration of anti-TNF-α agents is believed to interfere with this pathway by blocking the binding of tmTNF to TNFR2 and decreasing the production of IL-6 [36, 37]. As a result, pro-inflammatory cells regain their susceptibility to proapoptotic stimuli, which decreases the intensity of inflammatory infiltrates.

The immunoregulatory properties of OPRX-106 showed in patients with active UC confirmed previous observations made by the authors in animal models of chemically-induced steatohepatitis and colitis [38, 39]. They were able to show the reduction of inflammatory infiltrates after oral administration of OPRX-106, which was accompanied by the induction of regulatory T cells and the increase of anti-inflammatory cytokines. Whether these phenomena—described in animal models and humans—are mediated by the direct interaction of anti-TNF-α molecules with tmTNF on immune cells, as suggested in the case of parenterally administered TNF-α-blocking agents in IBD, is to be established. Nevertheless, the studies on the efficacy of OPRX-106 were the first to show not only the rationale for using orally administered anti-TNF-α molecules but also presented possible mechanistic explanations for their modes of anti-inflammatory action.

## Nanotechnology-based drug design and oral anti-TNF-α therapy: current knowledge, future directions

More recently, other attempts have been made to develop oral formulations of anti-TNF-α antibodies by using the discoveries of pharmaceutical nanotechnology. Kim et al. [40] proposed nanocomposite-based oral IFX delivery systems. All three designed liposomal drug formulations (liposome-coated IFX, aminoclay liposome-coated IFX, and Eudragit® S100 aminoclay liposome-coated IFX) showed a high encapsulation efficiency. A DSS murine model of colitis showed their capability to decrease intestinal inflammation on histomorphological and cytokine levels after oral administration. In line with this approach of using nanopharmaceuticals as a new formulation of mAbs, Wang et al. [41] discovered a nanoparticle based on natural polyphenol tannic acid and polyethylene glycol containing polymer for oral IFX. The solution of this novel pharmaceutical formula given as drinking water was very effective in achieving a high local concentration of therapeutic molecule directly in the inflamed intestinal tissues in a murine DSS-induced colitis model. Moreover, the authors showed that treatment with IFX-loaded nanoparticles ameliorated not only the inflammatory activity assessed histologically, but it also resulted in a decrease of serum inflammatory markers.

Table 2 summarizes the current achievements in the development of oral pharmaceutical formulations containing anti-TNF-α acting molecules.

**Table 2:** Summary of main pre-clinical and clinical studies with oral anti-tumor necrosis factor-α treatment in inflammatory bowel diseases

Authors (year of publication)	Anti-TNF-α-acting molecule	Pharmaceutical formulation	Type of the study	Main outcomes
Worledge et al. (2000) [26]	Avian anti-TNF-α antibody	Solution of polyclonal yolk IgY anti-TNF-α antibody diluted in carbonate buffer	Animal study (TNBS-induced colitis in rats)	Oral anti-TNF-α (600 mg/kg/day) therapy was significantly more effective in decreasing the colonic inflammatory activity assessed by gross morphology score, histology score, and tissue myeloperoxidase activity when compared to sulphasalazine (200 mg/kg/day) and dexamethasone (2 mg/kg/day)
Vandenbroucke et al. (2010) [27]	Monovalent and bivalent murine mTNF-neutralizing nanobody	Genetically modified *Lactococcus lactis* strain, secreting anti-mTNF nanobodies	Animal study (DSS-induced colitis and enterocolitis in IL-10^−/−^ mice)	Orally administered *L. lactis* secreting anti-mTNF nanobodies were effective in ameliorating experimental enterocolitis assessed histologically when compared with controls
Bhol et al. (2013) [75]	AVX-470—a polyclonal antibody specific for human TNF-α isolated from the colostrum of dairy cows	AVX-470 solution diluted in saline	Animal study (TNBS- and DSS-induced colitis in mice)	Orally administered AVX-470 significantly reduced colonic inflammation assessed endoscopically, histologically—comparably to prednisolone or parenteral etanercept, as well as decreased colonic expression of multiple pro-inflammatory proteins and mRNA levels of cytokines
Harris et al. (2016) [29] and Hartman et al. (2016) [28]	AVX-470—a polyclonal antibody specific for human TNF-α isolated from the colostrum of dairy cows	AVX-470-containing capsules	Randomized double-blind and placebo-controlled trial in patients with active moderate-to-severe ulcerative colitis	Oral administration of AVX-470 capsules for 4 weeks resulted in numerically higher percentages of patients achieving clinical, biochemical (CRP, IL-6), and endoscopic improvement when compared with placebo. AVX-470 also decreased the expressions of TNF and myeloperoxidase in the mucosa and diminished the apoptotic loss of epithelial cells. The therapy was safe and well-tolerated. No immunogenicity was detected
Maurer et al. (2016) [30] and Gareb et al. (2019) [31]	IFX	pH-sensitive ColoPulse tablets enabling drug release in the ileocolonic region	Gastrointestinal in vitro model (GISS) study, simulating gastrointestinal transit	ColoPulse-IFX tablets were stable in long-term storage conditions at room temperature and showed complete release in a simulated model of the ileocolonic region
Crowe et al. (2018) [32]	V565—a TNF-α-inhibiting antibody heavy chain variable domain	V565 solution loaded in a gastroprotective vehicle (NaHCO_3_ containing Marvel milk)	Animal study (DSS-induced colitis in mice) and in vitro study on human IBD tissue culture model	Oral administration of V565 led to a high drug concentration in colonic tissue and detectable drug serum levels in DSS-colitis mice. V565 decreased the production of proinflammatory cytokines to a similar extent as infliximab in ex vivo model of human IBD tissue
Crowe et al. (2019) [33]	V565—a TNF-α-inhibiting antibody heavy chain variable domain	Eudragit® enteric-coated V565 mini-tablets	Animal study (cynomolgus monkeys)	Enteric-coated V565 minitablets effectively transported the anti-TNF-α-acting molecule to the intestines, which was detected in the intestinal wall and faeces with a very low systemic exposure
Nurbhai et al. (2019) [34]	V565—a TNF-α-inhibiting antibody heavy chain variable domain	Eudragit® enteric-coated V565 mini-tablets	Human IBD study	Enteric-coated V565 minitablets were detected in ileal fluid and faeces of patients with IBD. After a 7-day oral therapy, V565 was detected in colonic biopsies and resulted in a decrease of tissue phosphoprotein levels, reflecting its anti-inflammatory properties. There were no serious adverse events (AE) or withdrawals due to AE
Kim et al. (2020) [40]	IFX	Nanocomposite formulations: IFX-L, AC-IFX-L, and EAC-IFX-L	Animal study (DSS-induced colitis in mice) and in vitro study on PBMC of IBD patients	Nanocomposites-based IFX oral therapy targeted to inflamed colonic tissues with minimal systemic exposure in animal models of IBD, leading to clinical and histomorphological improvement. The most significant improvement was seen in the case of AC-IFX-L, and EAC-IFX-L. These nanocomposite carriers loaded with IFX also significantly decreased the pro-inflammatory cytokine expression
Wang et al. (2020) [41]	IFX	IFX@PPNP given as drinking water	Animal study (DSS-induced colitis in mice)	The synthesis of IFX@PPNP was feasible. Oral administration of IFX@PPNP resulted in a high drug concentration locally in the inflamed intestines and low systemic exposure. Treatment with IFX@PPNP was highly effective in terms of clinical, histomorphological parameters, as well it led to a decrease in pro-inflammatory parameters in colonic tissue and serum
Almon et al. (2021) [35]	Recombinant TNFR2-Fc fusion protein (OPRX-106)	A lyophilized *Nicotiana tabacum* (BY2) tobacco plant expressing OPRX-106	Open-label clinical trial in patients with active mild-to-moderate ulcerative colitis	Oral administration of OPRX-106 for 8 weeks resulted in an almost 70% rate of clinical response. One-third of patients were in clinical remission. A decrease in fecal calprotectin and histologic activity was observed

*AC-IFX-L* aminoclay-liposome-coated infliximab, *AE* adverse events, *DSS* dextran sulfate sodium, *EAC-IFX-L* Eudragit® S100-aminoclay-liposome-coated infliximab, *GISS* gastrointestinal simulation system, *IBD* inflammatory bowel disease, *IFX* infliximab, *IFX@PPNP* polyphenol–polyethylene glycol-containing polymers self-assembled nanoparticles loaded with infliximab, *IFX-L* liposome-coated infliximab, *IL-10*^*−/−*^ interleukin 10-deficient, *mTNF* mouse tumor necrosis factor, *PBMC* peripheral blood mononuclear cells, *TNBS* 2,4,6-trinitrobenzene sulfonic acid

An ideal drug delivery system should combine the ability to overcome anatomical and biological barriers, selectively recognize the target sites through surface ligands, and be stable, biodegradable, and non-toxic [42]. As reflected by the recent studies by Kim et al. and Wang et al. [40, 41] it seems that clinical application of orally administered anti-TNF-α antibodies in IBD could be shortly possible by utilizing the recent advances in the development of nanopharmaceuticals. According to the newest discoveries in this area, it can be hypothesized that orally administered mAbs-loaded lipid nanoparticles (LNPs) would maximize the advantages of targeted therapy in IBD. This goal can be achieved by efficient local drug release in the inflamed areas of the gastrointestinal tract with low systemic exposure, resulting in an improved safety profile of anti-TNF-α antibodies and a low risk of developing ADAs. Moreover, LNPs have the potential to increase the stability of a loaded therapeutic molecule. This feature of LNPs allows overcoming another disadvantage of mAbs, which is the risk of partial drug degradation over storage time.

**Fig. 2:**
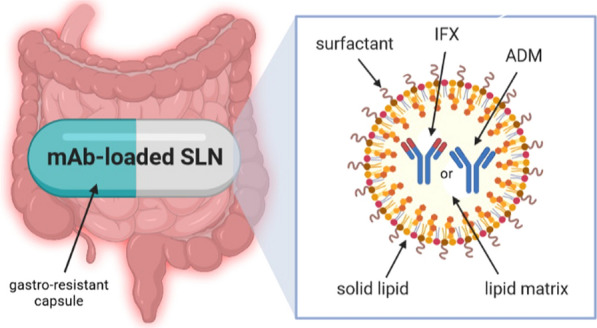
Schematic representation of SLN—loaded with antibody (own drawing). *mAb* monoclonal antibody, *SLN* solid lipid nanoparticles, *IFX* infliximab, *ADM* adalimumab

LPNs are obtained from biodegradable lipid materials of physiological nature and of high melting point. The high melting point (usually above room and body temperature) ensures that the lipid core of nanoparticles is solid, promoting the sustained release of the loaded drug [43]. The lipid character of these drug delivery systems is particularly suited for oral administration as they will undergo the same metabolic pathways as the lipids from food [17, 44]. Lipids work as absorption enhancers, thereby improving the bioavailability of the loaded drugs. This is particularly interesting for drugs belonging to classes II and IV of the Biopharmaceutical Classification System (BCS) [22]. The advantages of using LNPs as promising carriers for the oral administration of mAbs are attributed to their biodegradability, low cytotoxicity, high drug loading capacity, and scalability. Their production is cost-effective, and the particles provide a drug release in a controlled manner for up to several weeks [45]. Both types of LNPs, namely solid lipid nanoparticles (SLN) and nanostructured lipid carriers (NLC), are currently considered to be the newest and the most effective carriers of active substances [23, 46, 47]. Their main advantage is to increase the bioavailability of the incorporated drug administered by different routes [48, 49]. They can modify drug release [50] for site-specific targeting of the drug to improve its bioavailability [51, 52]. LNPs are composed of biodegradable and biocompatible lipids [53, 54], solid at room and body temperatures. They have also been successfully proposed to encapsulate proteins and small peptides [45, 46, 55–59]. All lipids and surfactants used for the synthesis of LNPs are classified by the Food and Drug Administration (FDA) and European Medicine Agency (EMA) as generally regarded as safe substances, of recognized biocompatibility and biodegradability since they are physiological lipids that occur naturally in the organism [53, 57]. The location of the drug in the lipid matrix governs its release rate [45], being dependent on the type and concentration of lipids, surfactants, and drug and on the selected production method [60]. SLN and NLC can occur in three different types of structures, defined either by the type of lipids used for their production or by the location of the drug in the lipid matrix [61, 62]. Loaded drugs can be placed between fatty acid chains or between lipid layers. SLN work as absorption enhancers when orally administered [62–64], while NLC increase loading capacity for drugs that usually show higher solubility in liquid lipids than in solid lipids (Fig. 2) [65, 66].

**Fig. 3:**
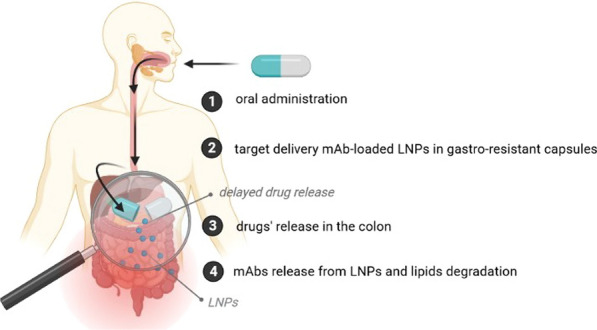
Graphically presented roadmap to a new therapeutic era of oral anti-TNF-α therapy (own drawing). *anti-TNF-α* anti-tumor necrosis factor-alpha, *mAb* monoclonal antibody, *LNPs* lipid nanoparticles

The use of nanoparticles for oral administration is mainly associated with the safety of LNPs and their ability to promote enteral absorption with the increased bioavailability of both hydrophilic and lipophilic drugs. On the other hand, understanding the impact of the size and shape of LNPs on their distribution in the intestine can be used to develop improved drug delivery systems to treat gastrointestinal diseases, such as IBD. The biodegradable lipid matrix of SLN/NLC undergoes enzymatic decomposition into components naturally occurring in the human body [53]. Due to the ability of LNPs to delayed drug release, SLN/NLC can be featured for site-specific, targeted, and modified-drug release for the treatment of inflammation in the course of IBD. It is worth underlining that a potential enteric formulation could be developed for the delayed release of the actives into the colon by encapsulating drugs-loaded LNPs in gastro-resistant capsules to prevent earlier degradation of nanoparticles in the stomach (Fig. 3).

Enteric-coated systems are intended to pursue colon delivery by exploiting differences in the pH of gastrointestinal fluids [67]. Loading the drug in the pH-sensitive polymers allows for delayed release by protecting the active ingredient from the acidic pH of the stomach and proximal small intestine. These polymers then break down in the more basic pH of the terminal ileum, thus providing a targeted drug delivery to the ileum and colon [68]. One of the most recommended pH-sensitive polymers in designing of ileocolonic-targeted drug delivery systems is methacrylic-acid-based polymers [67–70]. The polymethacrylates with a pH-dependent dissolution threshold ranging from pH 6.0 to 7.0 can be successfully used as coating agents, which protect the drug core against gastric juice and proximal small intestinal contents [67]. The results of conducted release studies have already proved that the Eudragit® enteric-coated matrix tablets successfully achieved gastric resistance and timed-release of the drug, assuring an adequate lag time for the intended ileocolonic targeting followed by a controlled-release phase [69, 70]. Therefore, this formulation strategy behind most anti-inflammatory drugs is commercially available worldwide for the therapy of IBD.

Besides the advantages of LNPs towards drug stability and bioavailability, they must take physicochemical interactions between carriers and loaded proteins (antibodies) into account, to ensure the release of intact and biologically active drugs. Drugs may be located inside the nanoparticles matrix or be adsorbed onto the surface of the nanoparticles. Thus, depending on their location, the antibody-lipid matrix interactions may distinctly affect antibody structure and bioactivity. The intermolecular forces between the protein and the lipid matrix may encompass covalent and electrostatic binding, polarization interaction, dispersion forces, and hydrophobic binding. However, there is a lack of knowledge on how these interactions may affect the 3D structure of proteins and, ultimately, how they influence bioactivity.

**Fig. 4:**
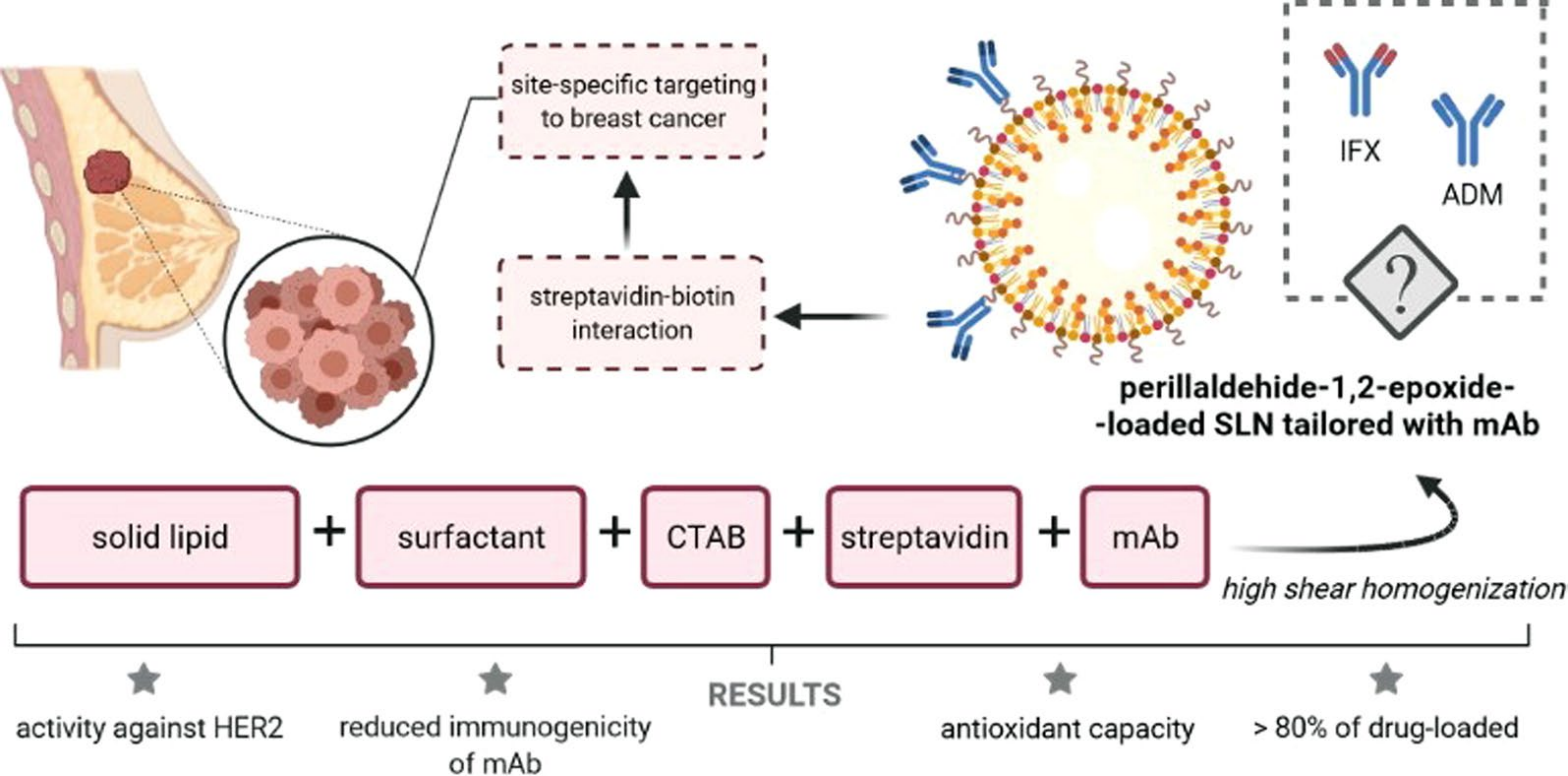
Graphically presented already developed SLN-tailored mAbs by the authors (own drawing). *CTAB* cetyltrimethylammonium bromide, *mAb* monoclonal antibody, *SLN* solid lipid nanoparticles, *HER2* human epithelial growth receptor 2, *IFX* infliximab, *ADM* adalimumab

Till now, several drug-designing protocols have been successfully finalized, and different nanoparticle-based therapeutic formulations containing monoclonal antibodies have been developed. As an example, our group has recently described a new cationic SLN formulation composed of solid lipid (Compritol ATO 888), surfactant (Poloxamer 188), and cetyltrimethylammonium bromide (CTAB) to incorporate perillaldehyde 1,2-epoxide, and surface-tailored with a mAb for site-specific targeting of human epithelial growth receptor 2 (HER2) [71]. Perillaldehyde 1,2-epoxide-loaded cationic SLN (cPa-SLN) were produced by high shear homogenization, achieving more than 80% of drug encapsulated in the lipid matrix. The study showed that the cytotoxic effect of perillaldehyde 1,2-epoxide against MCF-7 cell lines could be alleviated when surface-modifying the particles with streptavidin. The particles exhibited some antioxidant capacity attributed to the encapsulated monoterpene derivative. The cationic character of these particles provided a binding pathway via streptavidin to mAb. Streptavidin adsorption onto cPa-SLN-mAb improved the cell viability in comparison to the cationic cPa-SLN. The obtained results strengthen the potential use of mAb-coated lipid nanoparticles to increase mAb stability while reducing its immunogenicity. Cationic SLN have also been successfully tailored with a compact antibody against HER2 via streptavidin–biotin interaction to promote site-specific targeting to breast cancer cells [71]. We have found that streptavidin adsorption did not affect cell viability nor SLN accumulation in the target cells. Still, the surface-tailored SLN significantly improved cell internalization (with higher internalization in HER2/neu positive BT-474 than in HER2/neu negative MCF-7). At the same time, cytotoxicity was solely governed by the inherent toxicity profile of the lipid matrix (Fig. 4).

The concept of loading anti-TNF-α antibodies into novel orally administered formulations can also have some possible limitations—pharmaceutical and clinical. First of all, their administration through the oral route can be compromised by their stability in the gastrointestinal tract. Yadav et al. [72] have shown that proteolytic enzyme elastase was the main responsible for the instability of mAbs (e.g., infliximab and adalimumab) in the small intestine and to a lesser extent—the presence of trypsin and chymotrypsin. On the other hand, Wallace et al. [73] reported that mABs’ stability and susceptibility to proteases is governed by the gastrointestinal regions. Kim et al. [40] have shown that oral delivery systems tailored with mAbs can improve the bioavailability of low solubility and high permeability drugs. Loaded antibodies are less cytotoxic, thereby with the need of lower dosages of mAb, to achieve an efficient delivery and loading of macromolecules. It is known that the enteric methacrylic acid copolymer Eudragit® efficiently degrade and deliver drugs only at intestinal-specific pH both in vivo and in vitro, thus minimizing drug side effects [74]. Previous experiments performed by other authors showed that this concept is fully feasible and allows for effective oral delivery of therapeutic mAbs directly to the inflamed intestinal tissues without risk of premature proteolysis and denaturation [32, 33, 40]. That is why, based on the pre-clinical studies conducted so far, there is no current evidence on any chemical or pharmaceutical limitations regarding loading antibodies in novel nanocarriers. Therefore, oral anti-TNF-based nanocarriers are considered up-and-coming therapeutic approaches for treating IBD. They have shown a significant anti-inflammatory effect and remarkably decreased TNF-α levels in a DSS-induced mouse colitis model, as it was discussed above.

Regarding the possible clinical limitations, orally administered TNF-α inhibitors can be insufficient for IBD patients with the most severe forms of the disease. This can be due to a targeted mode of action directly in the gastrointestinal wall with considerably low systemic exposure. This could also be problematic in the case of patients experiencing extraintestinal manifestations. However, individuals with the highest IBD activity usually have to be hospitalized and are preferably treated using intravenous and/or subcutaneous drugs. In this scenario, starting oral anti-inflammatory treatment can be considered after the initial induction of clinical response via the parenteral route. On the other hand, irrespectively of the clinical circumstances, the activity of intestinal inflammation is the driving factor of all symptoms and complications typical for IBD. Thus, a rapid decrease in disease severity in the intestinal tissues induced by orally administered therapeutic molecules can also indirectly result in a systemic response. Therefore, it seems that oral administration of anti-TNF-α antibodies could be considered in the phase of inducing remission in IBD of mild-to-moderate activity and in selected patients with severe disease, as well as in all patients in the maintenance treatment.

## Conclusions

The main goal of IBD therapy is to treat the inflamed gastrointestinal tract to achieve steroid-free remission and to protect patients from irreversible bowel damage, together with a life-long disability. Among a growing range of therapeutic options, biologic agents, in particular TNF-α inhibitors, have revolutionized treatment strategies in IBD with the highest anti-inflammatory potential. Despite rapid advances in the quality of care in IBD brought by these biologic agents, there are still some significant limitations to consider. Currently, biologic agents are administered parenterally, which results in a systemic mode of action, particularly beneficial for those severely ill. However, this can also lead to immunogenicity and serious adverse events and often has to be performed under professional medical supervision.

**Fig. 5:**
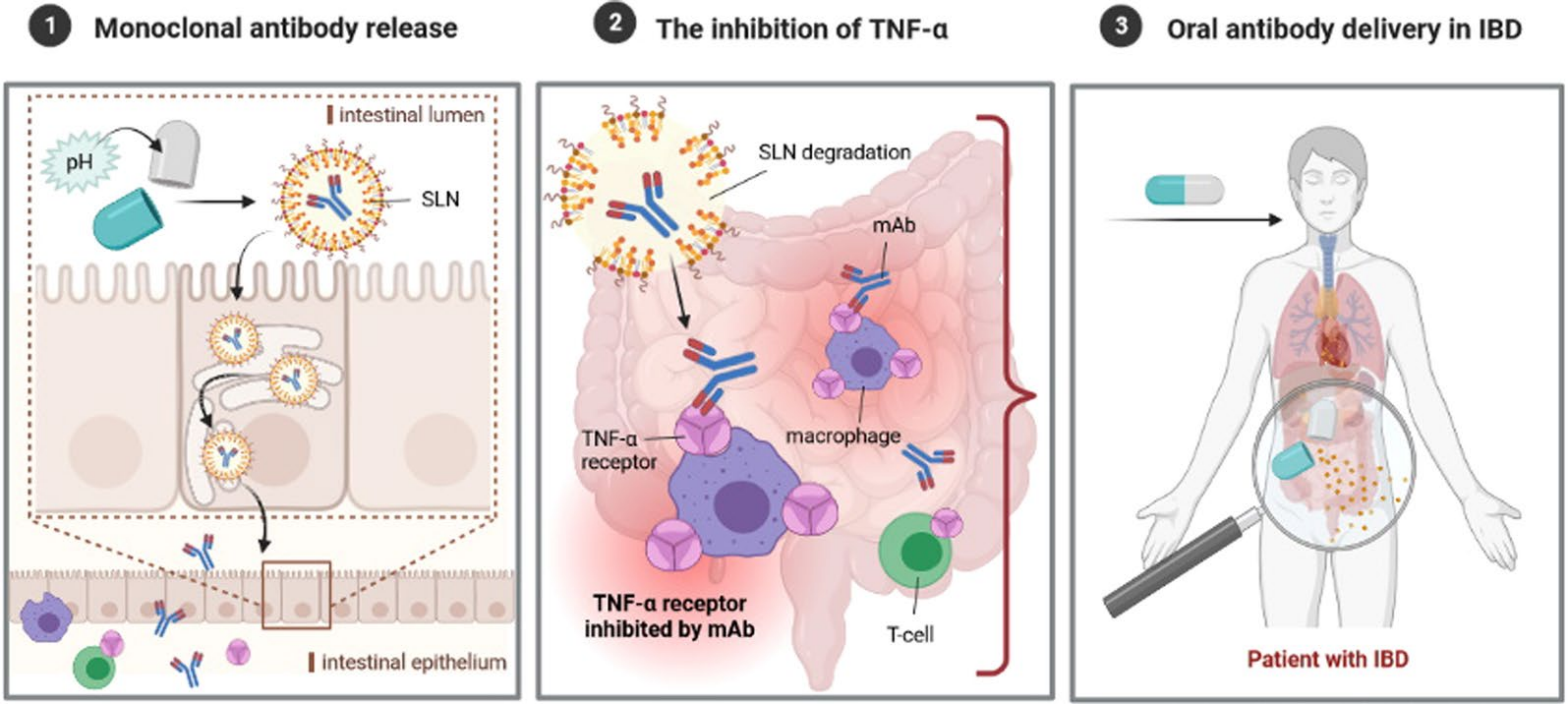
The summary of the concept of orally administered SLN-tailored anti-TNF-α mAb. After pH-dependent release from the capsule in the lumen of gastrointestinal tract, SLN undergoes degradation, which enables a direct interaction between the molecule and immune cells. As a result, an anti-inflammatory response is induced. *mAb* monoclonal antibody, *SLN* solid lipid nanoparticles

That is why it is hypothesized that introducing oral anti-TNF-α therapy can revolutionize treatment algorithms and significantly improve clinical outcomes in IBD. Several attempts have been made in this area. The promising results of a growing number of nanotechnology-based scientific protocols focused on developing orally administered formulations of nanoparticles loaded with mAbs are believed to enable entering a new era of orally administered biologic therapy. Great expectations are raised to the newly proposed pharmaceutical formulation of TNF-α inhibitors loaded in LNPs (Fig. 5). The advantages of LNPs as carriers for oral administration of mAbs are attributed to their biodegradability, low cytotoxicity, high drug loading capacity, and scalability. The production of LNPs is cost-effective, and the particles ensure drug release in a controlled manner for up to several weeks. LNPs can be designed and optimized to enable a modified drug release in the terminal ileum and colon, maximizing the bioavailability of mAbs. Such formulations should ensure high anti-inflammatory drug activity in an inflamed gut with a considerably low systemic exposure, resulting in lower immunogenicity and improved safety profile. Since oral drug delivery is considered the most convenient drug administration route with high patients compliance, this approach would significantly improve the quality of life of IBD patients who are otherwise bound to get regular TNF-α inhibitor injections. It could also open the door for the new potential biologic agents to be delivered orally in the future treatment of IBD.

## Data Availability

Not applicable.
